# Psychological Impact of COVID-19 Pandemic Among the General Population in Jordan

**DOI:** 10.3389/fpsyt.2021.618993

**Published:** 2021-04-14

**Authors:** Moawiah Khatatbeh, Aws Khasawneh, Hasan Hussein, Omar Altahat, Fadwa Alhalaiqa

**Affiliations:** ^1^Department of Basic Medical Sciences, Faculty of Medicine, Yarmouk University, Irbid, Jordan; ^2^Psychiatry, Neurosciences Department, Jordan University of Science and Technology and King Abdullah University Hospital, Irbid, Jordan; ^3^Psychiatric Mental Health Department, Faculty of Nursing, Philadelphia University, Amman, Jordan

**Keywords:** anxiety, COVID-19, mental health, psychological, stress

## Abstract

**Objective:** Pandemics are claimed to result in certain stressors. However, the potential psychological impact of a pandemic is often overlooked. The current study aimed to assess the psychological impact of the COVID-19 pandemic on Jordanians and to evaluate the influence of the socio-demographic variables on this impact.

**Method:** The current study employed a descriptive cross-sectional design using the Impact of Event Scale—Revised (IES-R) via a web-based questionnaire. The researchers utilized convenience sampling which led to a total of 2,854 participants from the 12 governorates of Jordan.

**Results:** The average score of the participants' responses on the IES-R questionnaire turned out to be 22.5 ± 11.7. Females were found to have more than double the odds of having an increased IES-R score [odds ratio (OR) = 2.2, confidence interval (CI) = 1.76–2.67] and participants who were older than 65 years had triple the odds of having the same risk compared to young adults aged 18–25 years (OR = 3.1, CI = 1.3–7.4). Significantly, having a family member diagnosed with COVID-19 placed individuals at a 7-fold higher risk of having an increased IRS-R score compared to their counterparts who did not have a family member diagnosed with COVID-19 (OR = 7, CI = 3.7–13.3).

**Conclusion:** COVID-19 pandemic has imposed significant level of psychological burden on Jordanians, especially among females. Governments should collaborate with psychiatrists, mental health professionals and local institutions to offer high-quality, timely crisis-oriented psychological services to the affected individuals for the duration of the COVID-19 pandemic.

## Introduction

Pandemics can result in certain stressors. However, potential excessive effects of the pandemic are often overlooked, especially when it comes to the psychological impact, which can be more pronounced and lasts longer than the purely somatic effects of the disease (1). Not only are these psychological effects significant in their ability to trigger mental health disorders such as anxiety, mood disorders, and post-traumatic stress disorder (PTSD), but they also play a role in the adherence to public health measures, such as vaccination and social distancing, and can result in socially disruptive behaviors such as civil unrest (2). Such a scenario is precisely the case during the COVID-19 pandemic (3).

The World Health Organization (WHO) declared the COVID-19 outbreak as an international public health emergency on 30 January 2020 (4) and a pandemic on 11 March 2020 (5). It has been claimed that certain subsets of the population might be vulnerable to the mental health impact of COVID-19, including healthcare workers (6), refugees (7), and older adults (8).

In Jordan, the first case of COVID-19 was confirmed at the beginning of March 2020, prompting the government to enforce strict quarantine measures after 2 weeks from confirming the first case and initiating a public information campaign utilizing various media to alert citizens about the dangers of the virus (9). The psychological impacts associated with such measures can be challenging to society and might be a source of distress (9, 10). This feeling can be provoked by the increased anxiety levels caused by earners' commitment to secure basic supplies (e.g., food, water, and medical supplies), given the financial loss due to losing jobs, fear of infection, and the stigma directed toward those who were quarantined after being in contact with COVID-19 confirmed cases (11, 12).

Previous research has demonstrated the capability of COVID-19 to increase rates of depression and anxiety in the population, especially among females (13). A cross-sectional study on a sample of 4,700 people in Istanbul, Turkey, utilizing the Fatigue Assessment Scale (FAS), found that 64.1% of the population was categorized as psychologically fatigued during the COVID-19 pandemic (14). In Jordan, using the Arabic version of the Depression, Anxiety and Stress Scale (DASS), the prevalence of depression, anxiety, and stress was found to be high among Jordanian nurses, and those who had close contact with COVID-19 patients showed stronger psychological reactions than other nurses who had not been in contact with COVID-19 patients (15).

Several studies have assessed the psychological impact of the pandemic using the Impact of Event Scale—Revised (IES-R). In China, a longitudinal study on a sample of 1,738 people reported that IES-R scores were higher than the cutoff score (≥24) for PTSD symptoms (13). Another study in Egypt, utilizing a cross-sectional design on a sample of 510 adults, found a mean IES-R score of 34.3 ± 15, with female gender and having chronic diseases considered as positive predictors for higher IES-R scores (16). On the other hand, a cross-sectional study of 1,160 respondents in Saudi Arabia obtained a mean IES-R score of 20.9 ± 15.7, which is below the cutoff score of ≥24 for PTSD symptoms, but still leaves 40.3% of the respondents with at least mild PTSD symptoms (17).

In Jordan, the psychological effects of COVID-19 infection are extremely noteworthy given the mental health and psychosocial context of the Jordanian community where stigma surrounding mental health, the general lack of mental health awareness, transportation issues and financial costs all serve as barriers for utilizing mental health services (18). To the best of the researchers' knowledge, this is the first study on the psychological impact of the COVID-19 pandemic among the general population of Jordan. Thus, the findings are likely to fill a gap in the existing literature and allow better comparability with other studies conducted in other regions using the same scale. It was hypothesized that COVID-19 pandemic has a significant burden of psychological impact on Jordanians and certain demographic characteristics could contribute to the level of this impact. Therefore, the purpose of this study was to assess the psychological impact of the COVID-19 pandemic on Jordanians and to evaluate the influence of the socio-demographic variables on this impact.

## Materials and Methods

### Study Design and Participants

This is a descriptive cross-sectional study targeting the general population of Jordan. A web-based questionnaire was designed for this study and distributed to those who could access the online form, utilizing convenience sampling. The form, along with a brief description of the study, was sent to potential participants via social media platforms such as Facebook and WhatsApp using the web link of the surveying questionnaire. Moreover, respondents were instructed to send the questionnaire link to their friends during the period from June to September, 2020. The inclusion criteria for participation were to be a Jordanian citizen, aged 18 years or older, able to understand and read Arabic and willing to complete the online survey. The exclusion criteria involved having a prior diagnosis of any psychiatric condition and not meeting any of the above-mentioned inclusion criteria. The survey's average completion time was about 10 min.

### Study Instrument

To assess the public's psychological response during the COVID-19 outbreak, researchers have reviewed the available literature and a questionnaire was developed that consisted of 29 questions distributed over two main sections. The first section consisted of seven questions on the participants' socio-demographic characteristics: age, gender, marital status, education, region of residence and having a family member or friend diagnosed with COVID-19. The second section, comprising 22 questions, utilized the Arabic version of the IES-R, which is a validated, self-administered questionnaire determining the extent of psychological impact caused by traumatic events and routine life stress. The questionnaire was computerized using Google Forms and distributed to participants via different social media networks and methods of electronic communication.

### Scoring of the IES-R

The IES-R consists of 22 items measuring three subscales: intrusion, avoidance and hyperarousal; the IES-R standardized scoring schema was utilized. Multiple-choice items were scored on a five-point Likert scale ranging from 0 to 4 (0, not at all; 1, a little bit; 2, moderately; 3, quite a bit; 4, extremely). The items were summed to yield a total score of 0–88 divided into four levels of psychological impact: normal (0–23); mild (19–27); moderate (28–31); severe (≥37) (32).

### Instrument Translation

The IES-R questionnaire was translated into Arabic by two, bilingual, independent professional translators with mental health expertise. The scale was then back-translated into English. The experts reviewed both versions and minor language changes were made while maintaining the content meaning.

### Validity and Reliability

The IES-R has been validated and widely used in the literature. However, two researchers checked its content and face validity before the final approval. To ensure its reliability, the IES-R was pilot-tested with the first 25 responses; based on these responses and the feedback, Cronbach's alpha score was 0.82. Similarly, Cronbach's alpha showed high internal consistency for the total scale (0.85) when considering the whole sample.

### Ethical Consideration

The study was designed according to the principles of the Declaration of Helsinki on research conduct. Informed consent was obtained from all participants by stating: “completing the questionnaire will be considered as consent to participate.” Approval to conduct the study was gained from the Faculty of Medicine at Yarmouk University, Jordan. To maintain the confidentiality and anonymity of their data, participants were informed that the research will not reveal any identifying information; data will be saved under lock and key with only the research team will have access to it.

### Statistical Analysis

SPSS Version 20.0 (IBM, Chicago, IL, USA) statistical software was used to analyse the data. Quantitative results were reported as either mean and standard deviation (mean ± SD) or frequency and percentage. A cross-tabulation analysis using the chi-square test was employed to assess if there were significant associations between categorical variables. Significant factors revealed from the cross-tabulation analysis were subjected to a backward Wald stepwise binary logistic regression analysis to assess the independent effect of each factor after controlling for potential confounders. A *p* value of < 0.05 was considered to be statistically significant.

## Results

The data were collected through online surveys. By utilizing convenience sampling, we received 3,136 responses from all governorates of Jordan. Of these, 368 (11.7%) incomplete responses and 28 (0.9%) responses for participants with prior psychological conditions were excluded, leaving 2,854 responses (87.4% of the total sample) valid for the final analysis. The majority of participants were young adults aged 18–25 years and about 89% of them had at least undergraduate degree. Detailed socio-demographic characteristics of the study population are shown in Table 1.

**Table 1:** Socio-demographic characteristics of the study population (*n* = 2854).

**Characteristic**	***n* (%)**
**Gender**	
Male Female	1,181 (41.4) 1,673 (58.6)
**Age (years)**	
18–25 26–35	1,641 (57.5) 507 (17.8)
36–45 46–55	330 (11.6) 261 (9.1
56–65 > 65	82 (2.9) 33 (1.2)
**Marital status**	
Single Married Widowed Divorced	1,859 (65.1) 942 (33.0) 22 (0.8) 31 (1.1)
**Education**	
Secondary or less Undergraduate Postgraduate	314 (11.0) 2,293 (80.3) 247 (8.7)
**Region of residence**	
North Central South	2,035 (71.3) 735 (25.8) 84 (2.9)
**Diagnosis of COVID-19 in the family**	
No Yes	2,794 (97.9) 60 (2.1)
**Diagnosis of COVID-19 in relatives or friends**	
No Yes	2,415 (84.6) 439 (15.4)

With a range of 0–88, the average score of the participants' responses on the IES-R questionnaire was 22.5 ± 11.7, denoting upper-normal stressful impact. More than half of the study sample had a normal IES-R score (56.9%). Furthermore, 23.3, 9.5, and 10.3% had mild, moderate, and severe IES-R scores, respectively. The average scores (mean ± SD) of the IES-R subscales were: intrusion, 6.1 ± 4.9; avoidance, 10.1 ± 5.4; hyperarousal, 6.2 ± 4.2.

To assess the impact of the socio-demographic characteristics on the IES-R score a cross-tabulation analysis was performed, revealing that all the factors were significantly correlated with the IES-R score apart from education level. As expected, having a family member, relative, or friend diagnosed with COVID-19 was strongly correlated with a higher IES-R score. The results of this analysis are shown in Table 2.

**Table 2:** Cross-tabulation of factors associated with the Impact of Event Scale – Revised score.

	**Total score categories**				***p***
	**Normal**	**Mild**	**Moderate**	**Severe**	
	***n* (%)**	***n* (%)**	***n* (%)**	***n* (%)**	
**Gender**					<0.001
Female	834 (49.9)	425 (25.4)	189 (11.3)	225 (13.4)	
Male	791 (67.0)	239 (20.2)	81 (6.9)	70 (5.9)	
**Age**					0.012
18–25	942 (57.4)	395 (24.1)	141 (8.6)	163 (9.9)	
26–35	304 (60.0)	105 (20.7)	49 (9.7)	49 (9.7)	
36–45	185 (56.1)	82 (24.80)	30 (9.10)	33 (10.0)	
46–55	137 (52.5)	59 (22.6)	30 (11.5)	35 (13.4)	
56–65	48 (58.5)	14 (17.1)	12 (14.6)	8 (9.8)	
+65	9 (27.3)	9 (27.3)	8 (24.2)	7 (21.2)	
**Marital Status**					0.013
Single	1,072 (57.7)	433 (23.3)	163 (8.8)	191 (10.3)	
Married	535 (56.8)	217 (23.0)	96 (10.2)	94 (10.0)	
Widow	5 (22.7)	6 (27.3)	6 (27.3)	5 (22.7)	
Divorced	13 (41.9)	8 (25.8)	5 (16.1)	5 (16.1)	
**Region of residence**					0.014
North	1,137 (55.9)	487 (23.9)	189 (9.3)	222 (10.9)	
Central	424 (57.7)	167 (22.7)	75 (10.2)	69 (9.4)	
Southern	64 (76.2)	10 (11.9)	6 (7.1)	4 (4.8)	
**Education**					0.270
≤ Secondary	170 (54.1)	89 (28.3)	22 (7.0)	33 (10.5)	
Graduate or diploma	1,317 (57.4)	517 (22.5)	226 (9.9)	223 (10.2)	
Postgraduate	138 (55.9)	58 (23.5)	22 (8.9)	29 (11.7%)	
**Diagnosis of COVID-19 in the family**					<0.001
Yes	0 (0.0)	14 (23.3)	23 (38.3)	23 (38.3)	
No	1,625 (58.2)	650 (23.3)	247 (8.8)	272 (9.7)	
**Diagnosis of COVID-19 in relatives or friends**					<0.001
Yes	104 (23.7)	153 (34.9)	71 (16.2)	111 (25.3)	
No	1,521 (63.0)	511 (21.2)	199 (8.2)	184 (7.6)	

In the binary regression analysis, the psychological impact was categorized into two main levels: normal (IES-R = 0–23) and abnormal (IES-R ≥ 24). The analysis revealed that gender, age and having a family member, relative or friend diagnosed with COVID-19 were predictors of a higher IES-R score. Females had more than double the odds of having a higher IES-R score [odds ratio (OR) = 2.2, confidence interval (CI) = 1.76–2.67]. Participants who were older than 65 years had triple the odds of having a higher IES-R score compared to young adults aged 18–25 years (OR = 3.1, CI = 1.3–7.4). Significantly, having a family member diagnosed with COVID-19 placed individuals at a 7-fold higher risk of having a higher IRS-R score compared to their counterparts who did not have a family member diagnosed with COVID-19 (OR = 7, CI = 3.7–13.3). Despite the insignificant statistical associations, being a widow, a divorced, and living in the Northern and central regions within Jordan constituted risk factors for an impaired psychological status. Table 3 presents the results of the binary logistic regression analysis.

**Table 3:** Logistic regression analysis of factors associated with an increased impact of event scale—revised score.

**Variable**	**Odds ratio**	**95% Confidence interval**		***P***
		**Lower**	**Upper**	
**Gender**				
Male	1^*^			
Female	2.2	1.76	2.67	0.001
**Age (years)**				
18–25	1^*^			
26–35	1.4	1.01	1.86	0.044
36–45	1.2	0.82	1.86	0.027
46–55	1.9	1.22	2.93	0.003
56–65	1.9	1.04	3.40	0.039
> 65	3.1	1.37	7.42	0.010
**COVID-19 diagnosis among family**				
No	1^*^			
Yes	7.0	3.76	13.38	0.001
**COVID-19 diagnosis in relatives or friends**				
No	1^*^			
Yes	2.9	2.31	3.73	0.001
**Marital status**				
Single	1^*^			
Married	1.1	0.90	1.34	0.332
Widow	2.9	1.83	6.01	0.017
Divorced	2.1	0.96	4.52	0.061
**Region of residence**				
North	2.0	1.02	3.93	0.075
Central	1.9	0.95	3.76	0.087
Southern	1^*^			

* *Reference for other categories*.

## Discussion

This study was aimed at assessing the psychological impact of the COVID-19 pandemic among the Jordanian general population and aimed at evaluating the association between this impact and participants' socio-demographic characteristics. It is well-established in the literature that infectious diseases can impose stressful situations. For example, during the Severe Acute Respiratory Syndrome (SARS) epidemic, about 16% of respondents from the general public in Hong Kong were found to have a moderate or severe stressful impact using the Chinese version of the IES (33). To that end, our study's results revealed an overall mean IES-R score of 22.5, which denotes a normal (upper level) psychological impact. This score is in agreement with results from Saudi Arabia where the general population reported a mean score of 20.9 using the IES-R (17). However, our IES-R score was less than those obtained from Egypt (16) and China (13, 34). This could be rationalized by the low incidence of COVID-19 in Jordan, and therefore, lower number of confirmed cases compared to other countries (9).

The lower IES-R scores in the current study could be attributed to the low prevalence in Jordan in the early months of the outbreak and the fact that the country succeeded in containing the spread of the disease during this period (9). Another possible explanation is that the disease outbreak was not considered as severe at the time when the study was conducted. The higher scores in China could be explained by the fact that China was the main focus of the pandemic. Furthermore, the higher scores among Egyptians could be referred to the perception of under-reporting of COVID-19 cases in Egypt (35).

The results of our study found that 19.8% of the respondents had a moderate or severe psychological impact of the COVID-19 outbreak. Similarly, 23.6% of the Saudi general population reported a moderate or severe psychological impact due to the pandemic (17).

In our study, avoidance was the most prevalent symptom among participants, revealing a score of 10.1 out of 32. This result is in agreement with results from Spain using the same IES-R (36). This may be due to the fact that almost all media and chats between individuals were overwhelmed by the pandemic news, its progression and that the prognosis and promises of vaccine discovery were poor. In such a situation, avoidance can improve mood and lower adverse thoughts. Additionally, avoidance is a normal defense mechanism that aims to avoid stress and reduce response if there is any loss of feeling. However, if it is adopted for long durations, it will have adverse consequences (e.g., not following the safety precautions, denial of the existence of the infection).

The female gender was significantly associated with a high level of IES-R. This finding is in line with similar results reported in Egypt (16), Saudi Arabia (17), Spain (36), China (19), and Italy (20). A recent systematic review has supported similar results (21). This is in line with previous studies which concluded that, after traumatic events, acute psychological disorders are more prevalent in females than males (22). This could be rationalized by the fact that women can better control their emotions in comparison to men (23).

Concerning the association between age and the psychological impact of COVID-19, older age was predictive of a higher IES-R score in our sample. Similar findings were reported in Saudi Arabia (24). Usually, chronic diseases, social isolation and loneliness are associated with older ages (25). These results are supported by the high prevalence of chronic diseases in Jordan (26), making these vulnerable individuals (usually older) more susceptible to COVID-19 infection (27). The WHO announced that having co-morbidities increases the risk of being infected with COVID-19. Therefore, in Jordan, stricter regulations were implemented concerning individuals older than 60 years during the pandemic. For example, they were instructed to stay at home and forbidden from attending public gatherings and places of worship. This situation can provoke the perception of increased susceptibility of those individuals and amplify their stressful feelings.

During the SARS outbreak, individuals who had one of their family members or friends quarantined or suspected of having the infection were reported to experience symptoms of depression (28). In our study, having relatives or acquaintances infected with COVID-19 was a risk factor for increasing the psychological impact among the population. This result is in line with results from a study in Italy which utilized a large sample size (18,147 participants) where having a loved one die by COVID-19 was associated with perceived stress, depressive symptoms, and insomnia (20). Similar findings were reported in other studies from Italy when individuals who were in contact with those infected by COVID-19 had an increased risk of developing sleep disturbances, as well as higher levels of anxiety and distress given that participants perceived COVID-19 pandemic a traumatic event (29–31). A similar trend was reported among university students in China (37). However, this result was not consistent with the results from Saudi Arabia obtained by Alkhamees et al. (17), who reported that having relatives diagnosed with COVID-19 seemed not to have a significant impact on the IES-R score. From the above, we can see the cultural diversity in reflecting the impact of COVID-19. Hence, culture must be taken into consideration in similar future studies.

Participants from the Northern and central areas in Jordan have shown higher IES-R scores compared to those from the Southern region. The number of cases in the Southern region was far less than that in other regions in the country at the time of conducting the study, which may contribute to less perceived risk among people in this region. This result is in agreement with results from China, where individuals from the general populations had lower IES-R scores when they perceived a low risk of catching COVID-19 infection (19). Similarly, in Italy, proximity to areas with higher prevalence of COVID-19 infection was associated with higher levels of psychological impact (20).

This study provides a portrait of the psychological well-being of the Jordanian population during the COVID-19 outbreak. A global response is urgently needed to help health systems deal with the problems caused by the COVID-19 pandemic. The findings of the current study from Jordan as a developing country, along with the results of previous studies, showed that the COVID-19 pandemic can lead to psychopathological symptoms such as stress, anxiety, and PTSD. These preliminary findings can be useful to predispose early interventions aimed at early detecting, diagnosing, and treating any COVID-19-associated psychological conditions of the population.

## Conclusion

Public health emergencies can drastically affect individuals' psychological health, which requires the attention, help and support of society and families. The average score of the participants' responses on the IES-R questionnaire was 22.5, representing an upper–normal stressful impact. This constitutes an alarming sign, especially after the significant resurgence and spread of COVID-19 in September 2020, which may lead to an increase in psychological problems among Jordanians.

## Recommendations

We recommend the provision of targeted high-quality, timely, crisis-oriented psychological interventions for communities affected for the duration of the COVID-19 pandemic. Special attention to vulnerable high-risk groups, including older adults and frontline workers, is crucial. Furthermore, enhanced awareness and diagnosis of mental disorders at the primary care level and improved access to psychological interventions (especially those delivered through smart platforms) are needed. Therefore, the government should collaborate with psychiatrists, mental health professionals, and local institutions to start public psychological awareness campaigns in cooperation with media, psychiatric, and mental health specialists to promote mental health well-ness and secure reliable delivery methods for psychological interventions, especially for the high-risk groups, given the mental health and psychosocial context of Jordan where many barriers already limit the utilization of mental health services.

## Strengths and Limitations

This study has several strengths and limitations. Among the strengths, the large sample size (2,854 respondents) allowed us to perform a rigorous analysis and extract detailed results and associations. Furthermore, this is the first study to use the IES-R in Jordan and its use in other countries, has allowed us to compare our situation with that in those countries, thus providing valuable information about the current situation that is useful in gaining insight about similar scenarios in the future.

Concerning the limitations, this was a cross-sectional study conducted during an unprecedented situation. The snapshot effect of this research design prevents it from ascertaining how mental health indicators are changing according to changes in the spread of COVID-19 and associated interventions. Longitudinal studies are needed to analyse the long-term impact of the pandemic on the individuals' psychological status and draw cause-and-effect relationships between dependent and independent variables. Additionally, the current study used a convenience sampling technique, which led to oversampling of Jordan's northern region; this may contribute to some limitations in generalizing its results. To be able to generalize findings, multicenter, and larger-scale surveys should be conducted. Furthermore, this study used an online platform questionnaire, which imposes selection bias of participants and might preclude those who have strong feelings about the issue under investigation or those who are less technologically literate from accessing the questionnaire. Therefore, clinical variables may not be entirely reliable. These limitations reduce the representativeness of our findings and may have influenced the results of the study. However, the adoption of an online survey was the best solution in this emergency where social distancing measures limited the data collection.

## Data Availability Statement

The raw data supporting the conclusions of this article will be made available by the authors, without undue reservation.

## Ethics Statement

This study was reviewed and approved by the Research Ethics Committee-Yarmouk University. All participants provided their written informed consent to participate in this study.

## Author Contributions

MK, FA, and AK: conceptualization. MK, FA, HH, and OA: methodology. MK and FA: formal analysis. HH and OA: data curation. MK, FA, AK, HH, and OA: writing—original draft preparation and writing—review and editing. MK: project administration. All authors read and agreed on the final version of the manuscript.

## Conflict of Interest

The authors declare that the research was conducted in the absence of any commercial or financial relationships that could be construed as a potential conflict of interest.

## References

[1] Pillay AL Barnes BR. Psychology and COVID-19: impacts, themes and way forward. South African J Psychol. (2020) 50:148–53. 10.1177/0081246320937684

[2] Taylor S. The Psychology of Pandemics: Preparing for the Next Global Outbreak of Infectious Disease. Newcastle upon Tyne: Cambridge Scholars Publishing (2019).

[3] Cullen W Gulati G Kelly BD. Mental health in the COVID-19 pandemic. QJM. (2020) 113:311–2. 10.1093/qjmed/hcaa110 32227218 PMC7184387

[4] Sohrabi C Alsafi Z O'Neill N Khan M Kerwan A Al-Jabir A. World Health Organization declares global emergency: a review of the 2019 Novel Coronavirus (COVID-19). Int J Surg. (2020) 76:71–6. 10.1016/j.ijsu.2020.02.034 32112977 PMC7105032

[5] World Health Organization (2020). WHO Director-General's Opening Remarks at the Media Briefing on COVID-19 – 11 March 2020. Retrieved from: https://www.who.int/director-general/speeches/detail/who-director-general-s-opening-remarks-at-the-media-briefing-on-covid-19---11-march-2020 (accessed March 11, 2020).

[6] Spoorthy MS Pratapa SK Mahant S. Mental health problems faced by healthcare workers due to the COVID-19 pandemic: a review. Asian J Psychiatr.(2020) 51:102119. 10.1016/j.ajp.2020.102119 32339895 PMC7175897

[7] El-Khatib Z Al Nsour M Khader YS Abu Khudair M. Mental health support in Jordan for the general population and for the refugees in the Zaatari camp during the period of COVID-19 lockdown. Psychol Trauma. (2020) 12:511–4. 10.1037/tra0000813

[8] Yang Y Li W Zhang Q Zhang L Cheung T Xiang YT. Mental health services for older adults in China during the COVID-19 outbreak. Lancet Psychiatr. (2020) 7:e19. 10.1016/S2215-0366(20)30079-1 32085843 PMC7128970

[9] Khatatbeh M. Efficacy of nationwide curfew to encounter spread of COVID-19: a case from Jordan. Front Public Health. (2020) 8:394. 10.3389/fpubh.2020.00394 32984234 PMC7475701

[10] Al-Tammemi AB. The battle against COVID-19 in Jordan: an early overview of the Jordanian experience. Front Public Health. (2020) 8:188. 10.3389/fpubh.2020.00188 32574291 PMC7220996

[11] Brooks SK Webster RK Smith LE Woodland L Wessely S Greenberg N Rubin GJ. The psychological impact of quarantine and how to reduce it: rapid review of the evidence. Lancet. (2020) 395:912–20. 10.1016/S0140-6736(20)30460-8 32112714 PMC7158942

[12] Galea S Merchant RM Lurie N. The mental health consequences of COVID-19 and physical distancing: the need for prevention and early intervention. JAMA Int Med. (2020) 180:817–8. 10.1001/jamainternmed.2020.1562 32275292

[13] Wang C Pan R Wan X Tan Y Xu L McIntyre RS. A longitudinal study on the mental health of general population during the COVID-19 epidemic in China. Brain Behav Immun. (2020) 87:40–8. 10.1016/j.bbi.2020.04.028 32298802 PMC7153528

[14] Morgul E Bener A Atak M Akyel S Aktaş S Bhugra D. COVID-19 pandemic and psychological fatigue in Turkey. Int J Soc Psychiatr. (2020) 10:0020764020941889. 10.1177/0020764020941889 32650681 PMC7355205

[15] Al-Amer R Malak MZ Aburumman G Darwish MM Nassar MS Darwish M Randal S. Prevalence and correlates of psychological reactions among Jordanian nurses during the coronavirus disease 2019 pandemic. Research Square. (2020). 10.21203/rs.3.rs-35820/v1

[16] El-Zoghby SM Soltan EM Salama HM. Impact of the COVID-19 pandemic on mental health and social support among adult Egyptians. J Commun Health. (2020) 45:689–95. 10.1007/s10900-020-00853-5 32468155 PMC7255077

[17] Alkhamees AA Alrashed SA Alzunaydi AA Almohimeed AS Aljohani MS. The psychological impact of COVID-19 pandemic on the general population of Saudi Arabia. Comprehensive Psychiatr. (2020) 102:152192. 10.1016/j.comppsych.2020.152192 PMC7354380 32688022

[18] International Medical Corps. Understanding the Mental Health and Psychosocial Needs, and Service Utilization of Syrian Refugees and Jordanian Nationals. A Qualitative and Quantitative Analysis in the Kingdom of Jordan. (2017). Retrieved from: https://data2.unhcr.org/en/documents/download/62036 (accessed 15 September 2020).

[19] Wang C Pan R Wan X Tan Y Xu L Ho CS. Immediate psychological responses and associated factors during the initial stage of the 2019 coronavirus disease (COVID-19) epidemic among the general population in China. Int J Environ Res Public Health. (2020) 17:1729. 10.3390/ijerph17051729 32155789 PMC7084952

[20] Rossi R Socci V Talevi D Mensi S Niolu C Pacitti F. COVID-19 pandemic and lockdown measures impact on mental health among the general population in Italy. Front Psychiatr. (2020) 11:790. 10.3389/fpsyt.2020.00790 32848952 PMC7426501

[21] Xiong J Lipsitz O Nasri F Lui LM Gill H Phan L. Impact of COVID-19 pandemic on mental health in the general population: a systematic review. J Affect Disord. (2020) 77:55–64. 10.1016/j.jad.2020.08.001 32799105 PMC7413844

[22] McLean CP Anderson ER. Brave men and timid women? A review of the gender differences in fear and anxiety. Clin Psychol Rev. (2009) 29:496–505. 10.1016/j.cpr.2009.05.003 19541399

[23] McRae K Ochsner KN Mauss IB Gabrieli JJ Gross JJ. Gender ifferences in emotion regulation: an fMRI study of cognitive reappraisal. Group Processes Int Relat. (2008) 11:143–62. 10.1177/1368430207088035 29743808 PMC5937254

[24] Almofada SK Alherbisch RJ Almuhraj NA Almeshary BN Alrabiah B Al Saffan A. Knowledge, attitudes, and practices toward COVID-19 in a Saudi Arabian population: a cross-sectional study. Cureus. (2020) 12:e8905. 10.7759/cureus.8905 32637289 PMC7331923

[25] Courtin E Knapp M. Social isolation, loneliness and health in old age: a scoping review. Health Soc Care Commun. (2016) 25:799–812. 10.1111/hsc.12311 26712585

[26] Mokdad A. Global non-communicable disease prevention: building on success by addressing an emerging health need in developing countries. J Health Specialt. (2016) 4:92. 10.4103/1658-600X.179820

[27] Tsai J Gelberg L Rosenheck RA. Changes in physical health after supported housing: results from the collaborative initiative to end chronic homelessness. J General Int Med. (2019) 34:1703–8. 10.1007/s11606-019-05070-y 31161570 PMC6712193

[28] Ko CH Yen CF Yen JY Yang MJ. Psychosocial impact among the public of the severe acute respiratory syndrome epidemic in Taiwan. Psychiatr Clin Neurosci. (2006) 60:397–403. 10.1111/j.1440-1819.2006.01522.x 16884438

[29] Casagrande M Favieri F Tambelli R Forte G. The enemy who sealed the world: effects quarantine due to the COVID-19 on sleep quality, anxiety, and psychological distress in the Italian population. Sleep Med. (2020) 75:12–20. 10.1016/j.sleep.2020.05.011 32853913 PMC7215153

[30] Forte G Favieri F Tambelli R Casagrande M. The enemy which sealed the world: effects of COVID-19 diffusion on the psychological state of the Italian population. J Clin Med. (2020) 9:1802. 10.3390/jcm9061802 32531884 PMC7356935

[31] Forte G Favieri F Tambelli R Casagrande M. COVID-19 pandemic in the italian population: validation of a post-traumatic stress disorder questionnaire and prevalence of PTSD symptomatology. Int J Environ Res Public Health. (2020) 17:4151. 10.3390/ijerph17114151 32532077 PMC7312976

[32] Beck JG Grant DM Read JP Clapp JD Coffey SF Miller LM. The impact of event scale-revised: psychometric properties in a sample of motor vehicle accident survivors. J Anxiety Disord. (2008) 22:187–98. 10.1016/j.janxdis.2007.02.007 17369016 PMC2259224

[33] Lau JT Yang X Tsui HY Pang E Wing YK. Positive mental health-related impacts of the SARS epidemic on the general public in Hong Kong and their associations with other negative impacts. J Infect. (2006) 53:114–24. 10.1016/j.jinf.2005.10.019 16343636 PMC7132442

[34] Zhang Y Ma ZF. Impact of the COVID-19 pandemic on mental health and quality of life among local residents in Liaoning Province, China: a cross-sectional study. Int J Environ Res Public Health. (2020) 17:2381. 10.3390/ijerph17072381 32244498 PMC7177660

[35] Medhat MA El Kassas M. COVID-19 in Egypt: uncovered figures or a different situation? J Global Health. (2020) 10:010368. 10.7189/jogh.10.010368 32566159 PMC7303805

[36] Rodríguez-Rey R Garrido-Hernansaiz H Collado S. Psychological impact of COVID-19 in Spain: early data report. Psychol Trauma. (2020) 12:550. 10.1037/tra0000943 32538657

[37] Cao W Fang Z Hou G Han M Xu X Dong J. The psychological impact of the COVID-19 epidemic on college students in China. Psychiatr Res. (2020) 287:112934. 10.1016/j.psychres.2020.112934 32229390 PMC7102633

